# Editorial: Cellular and molecular mechanisms of synaptic plasticity at hippocampal and cortical synapses

**DOI:** 10.3389/fncel.2022.980623

**Published:** 2022-07-15

**Authors:** Nathalia Vitureira, Roberto De Pasquale, Ricardo M. Leão, Francesco Mattia Rossi

**Affiliations:** ^1^Laboratorio de Comunicación Sináptica, Departamento de Fisiología, Facultad de Medicina, Universidad de la República, Montevideo, Uruguay; ^2^Department of Physiology and Biophysics, Institute of Biomedical Sciences, University of São Paulo, São Paulo, Brazil; ^3^Department of Physiology, School of Medicine of Ribeirão Preto, University of São Paulo, Ribeirão Preto, Brazil; ^4^Laboratorio de Neurociencias, Unidad de Neuroplasticidad, Facultad de Ciencias, Universidad de la República, Montevideo, Uruguay

**Keywords:** long-term potentiation (LTP), long-term depression (LTD), homeostatic synaptic plasticity (HSP), spike timing-dependent plasticity (STDP), neurological disease

One of the most fascinating properties of the nervous system is the ability to modify its structure and function in order to adapt to the continuously changing environment. The efficiency of the communication between two neurons is modulated by the plasticity of synapses, the strengthening or weakening over time of their function in response to changes in synaptic activity. The idea that neuronal circuits could change in an activity-dependent manner was first proposed by Donald Hebb in 1949. Nowadays, plasticity processes have been identified in almost all species, in primary modalities but also in multimodal functions. It is also well-established that plastic changes can span along different timescales and are required for the correct development of the nervous system as well as for experience-dependent memory and learning. Moreover, many neurological diseases and neuropsychiatric disorders are today interpreted as alterations in plasticity mechanisms.

The main objective of this Research Topic is to collect research articles and reviews to provide new insights and explanatory models capable of accounting for the complexity of the plasticity mechanisms used by the hippocampus and cerebral cortex to modulate the strength of neuronal circuits under physiological and pathological conditions. Based on the articles and reviews detailed below, the multi-level analysis becomes relevant to support our understanding of synaptic plasticity in the brain.

Vannini et al., use different methodological approaches (a combination of photoconversion and electron microscopy to assess changes of synaptic vesicle pools *in vivo*, electrophysiology and proteomics) to identify at nanoscale level alterations of homeostatic synaptic plasticity (HSP) mechanisms. They took advantage of a well-characterized model of chronic, focal epilepsy in the visual cortex of the mouse. Their data contribute to the characterization of the complex release machinery and the molecular modifications promoted by epileptic networks.

Chen et al., focus on a very relevant aspect of synaptic plasticity, which is the dynamic trafficking of AMPARs into and out of the synaptic membrane. In their work, they focus on Spastin, a microtubule-severing protein, whose mutations are considered the most common cause of hereditary spastic paraparesis. By using functional, molecular and biochemical strategies in hippocampal cultures they were able to better characterize the role of Spastin in AMPAR trafficking and advance our understanding of the synaptic plasticity and cognitive dysfunction underlying this pathology.

Cui et al., deepen the analysis at molecular level of the role of Rac1, a small GTPase of the Rho family, on the induction and maintenance of long-term potentiation (LTP) in the rodent hippocampus. By means of electrophysiological methods combined with biochemical and pharmacological approaches, they were able to show that during different stages of LTP, the activation of Rac1 can modulate different signaling pathways (activation of PKCι/λ by PI3K, and inhibition of PKMζ by LIMK), which leads to an opposing effect on the induction and maintenance of LTP in the hippocampus.

Chaloner and Cooke, based on previous research on long-lasting stimulus-selective response potentiation (SRP) in the primary mouse visual cortex, the authors explore the neocortical processes of plasticity occurring during habituation at distinct timescales. Using *in vivo* recordings and genetic manipulations, they show that cortical plasticity accompanying behavioral habituation occurs across seconds, minutes, and days of repeated stimulus experience. Moreover, they characterized the role of NMDA receptors and parvalbumin-positive interneurons in such processes, identifying a range of mechanistically separable forms of plasticity occurring at different timescales in the same learning mouse.

dos Santos Cardoso et al., focus on the analysis of the potentially beneficial effect of photobiomodulation (transcranial near-infrared laser treatment) on the aging brain. By investigating the expression and activation of distinct intracellular signaling proteins in the cerebral cortex and hippocampus of aged rats treated with the transcranial near-infrared laser, they identify that this experimental approach improves intracellular signaling pathways linked to cell survival, memory, and glucose metabolism.

Reyes-García and Escobar provide an overview of the experimental evidence supporting the relationship between long-term depression (LTD) and synaptic depotentiation with extinction in different models and summarized the established cellular and molecular mechanisms underlying this process. They discuss the role of calcineurin in the association between hebbian and HSP during new learning or re-learning processes.

Taylor and Jeans discuss the experimental evidence linking deficits in HSP with the onset and/or progression of major neurodegenerative diseases and describe the contribution of different HSP-associated proteins in neurodegeneration. By summarizing evidence obtained mainly in studies on Alzheimer, Parkinson, and Huntington disease and on amyotrophic lateral sclerosis, they suggest a distinct role of HSP in each of these major diseases associated with neurodegeneration.

Inglebert and Debanne review the relevance of physiological concentrations of extracellular Ca^2+^ in spike timing-dependent plasticity (STDP). They discuss experimental data and mathematical models that address the requirement of postsynaptic Ca^2+^ entry for the induction and/or maintenance of this form of long-term plasticity. They open the debate regarding whether synaptic plasticity rules inferred from *in vitro* studies could be applied to *in vivo* conditions and, in this sense, they question if different forms of STDP persist under physiological concentrations of Ca^2+^. They finally sum up a variety of experimental data, obtained from different animal models exploring the rules of STDP *in vivo*.

Meza et al., present a quite exhaustive revision of the role of a particular complex channel, the Transient Receptor Potential Vanilloid 1 (TRPV-1), in the modulation of synaptic function in different brain regions. The authors emphasized the main mechanisms described for the plasticity-related role of TRPV-1 at the pre and postsynaptic level, in glial cells, in interaction with the endocannabinoid system, development, mental disorders and neurological diseases such as epilepsy, anxiety, and depression, as well in drug-addiction disorders.

Ruggiero et al., focus on the relevance of the main neurotransmitter systems (acetylcholine, dopamine, noradrenaline, serotonin, and endocannabinoids) in long and short-term synaptic plasticity in the hippocampus-prefrontal cortex (HPC-PFC) pathway. In this comprehensive review they also discuss the implications of HPC-PFC disruption in synaptic plasticity and functional connectivity and thus in neuropsychiatric disorders, such as schizophrenia, major depression and anxiety, and in Alzheimer disease.

## Manuscript contribution

Neuronal plasticity is a fundamental aspect in the whole nervous system functioning. We believe that the manuscripts integrating this Research Topic contributed to a deeper characterization of the extremely complex mechanisms regulating plasticity in its different forms, in both physiological and pathological conditions.

## Author contributions

All authors listed have made a substantial, direct, and intellectual contribution to the work and approved it for publication.

## Conflict of interest

The authors declare that the research was conducted in the absence of any commercial or financial relationships that could be construed as a potential conflict of interest.

## Publisher's note

All claims expressed in this article are solely those of the authors and do not necessarily represent those of their affiliated organizations, or those of the publisher, the editors and the reviewers. Any product that may be evaluated in this article, or claim that may be made by its manufacturer, is not guaranteed or endorsed by the publisher.

